# Inorganic Hybrid Materials with Encapsulated Polyoxometalates

**DOI:** 10.3390/ma3010682

**Published:** 2010-01-25

**Authors:** Véronique Dufaud, Frédéric Lefebvre

**Affiliations:** 1 Laboratoire de Chimie, Université de Lyon, UMR 5182 CNRS-ENS Lyon, 46 Allée d'Italie, F-69364 Lyon cedex 07, France; 2 Laboratoire de Chimie, Catalyse, Polymères et Procédés, Université de Lyon, UMR 5265 CNRS-CPE Lyon, 43 bd du 11 Novembre 1918, 69622 Villeurbanne cedex, France

**Keywords:** polyoxometalates, encapsulation, inorganic hybrid material, nanocomposites

## Abstract

This review describes the synthesis and characterization of inorganic materials containing polyoxometalates encapsulated in oxide matrices. Examples illustrating key aspects in terms of synthesis and applications are presented according to the nature of the final hybrid material: those based on non-structured silicas, on mesostructured silicas, on macrostructured silicas and on other oxides. In each part, key points of the synthetic protocols are highlighted and structural features and properties of the resultant hybrid nanocomposites are discussed.

## 1. Introduction

Polyoxometalates (POMs) are a class of molecularly defined inorganic metal-oxide clusters formed from early transition metals (V, Nb, Ta, Mo, W) in their highest oxidation states [1]. Initially, studies were purely fundamental, without any practical applications in mind, but more recently numerous applications have appeared. POMs are attractive compounds due to their molecular and electronic structural diversity. Applications have been reported in diverse domains including medicine, material science, photochemistry or catalysis [2,3,4,5]. In catalysis, polyoxometalate compounds have been extensively used as acid and oxidation catalysts in many reactions, notably because their acid-base and redox properties can be tuned by simply changing the polyanion chemical composition. Unfortunately, these compounds are highly soluble in various polar solvents which renders them less attractive for applications in heterogeneous catalysis as their use with polar molecules will result in non-negligible leaching and thus deactivation of the catalytic system.

**Figure 1:**
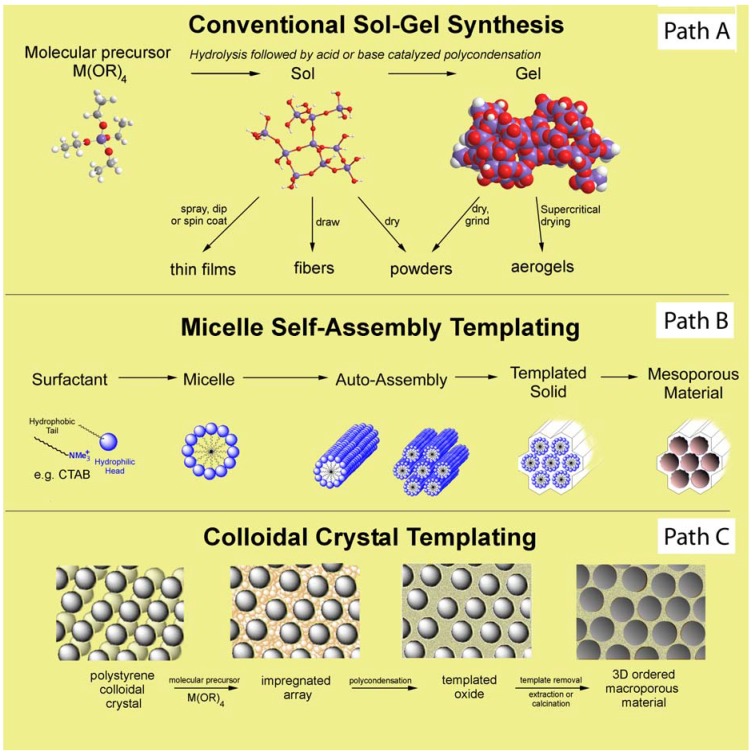
Common pathways to porous inorganic materials: (a) Conventional sol-gel route (b) Micelle self-assembly templating (c) Colloidal crystal templating.

Porous oxidic materials constitute an interesting class of solids widely used for applications in the fields of material science, adsorption and catalysis, due notably to their rigidity, thermal stability and regular, adjustable nanosized porous structure. In particular, porous materials with high surface area and regular geometry represent suitable supports for the immobilization of catalytically relevant species. Two major strategies have been described in the literature to achieve immobilization of POMs onto porous oxides: i) post-synthetic procedures involving the modification of the inner surface of a pre-formed oxide; the functionalization is, in this case, usually carried out by using the incipient wetness technique (also referred to as impregnation); ii) direct synthesis (also referred to as one-pot synthesis) implying the co-incorporation of an oxide precursor together with POM clusters using a sol-gel technique. The conventional post-synthesis impregnation method is undoubtedly the simplest route to access functionalized materials, but it suffers from several drawbacks including difficulty in achieving high POMs loading without significant decrease in surface area and ordering, loss of the initial high dispersity of supported POM units via leaching, and loss of homogeneity due to minor changes in the structure. All of these lead to reduced activity or stability of the immobilized POMs. On the contrary, the preparation of POM-oxide materials via direct synthesis leads to materials with larger pore sizes without pore blockage and more stable and uniform distribution of the active phase in the solid which in turn can enhance the performance of the resulting nanocomposites. In this review, we chose to focus on the main synthesis methodologies to entrap POMs clusters inside porous oxide frameworks with an emphasis on silica-based oxides. Our purpose is not to provide an exhaustive catalogue of all the works in this domain, but rather to illustrate through representative examples different applications. A common aspect of all the syntheses is the use of the sol-gel approach which can be combined with different supramolecular templating techniques (Figure 1). The review is organized into four parts based on the nature of the final hybrid material: those based on non-structured silicas, on mesostructured silicas, on macrostructured silicas and on other oxides. In each part, we will present the key points of the synthetic protocols and discuss the structural features and properties of the different types of hybrid nanocomposites obtained. We conclude with an example where the POM is integrated with both organic and inorganic matrices in the sophisticated synthesis of metal nanoparticules, mirroring the broader current trend in catalyst design toward nanometric or molecular scale structural control of active sites and their surroundings.

## 2. Non-Structured Silica Based Materials

The sol-gel technique is a method of choice for synthesizing porous silica-based materials. This soft inorganic chemistry process consists in inorganic polymerization of silica precursors (silicon alkoxides or silicates) in aqueous or alcoholic solutions giving rise to highly cross-linked solids (Figure 1, path A). This mild solid synthesis can be carried out over an extremely wide range of experimental conditions which in turn will govern the characteristics of the resulting material: for example cross-link density, porosity and homogeneity of the functional sites will depend on the pH of the solution, the method of solvent removal, the type of catalysis applied for the polycondensation reaction (acidic, basic, nucleophilic). In general, POMs containing silica composites were prepared by hydrolysis of tetraethyl orthosilicate (TEOS) in the presence of POM-ion at acidic pH, pH~2.0, which may be adjusted to avoid the decomposition of the POM moiety. POM-ion was entrapped into the silica network during TEOS polycondensation. Although this approach seems to be straightforward, subtle changes in the synthetic protocols may have a profound influence on the resulting material properties and thus considerable effort has been devoted to optimizing syntheses.

The pioneering studies in this field started with the attempts of Izumi *et al.* to render the acidic cesium salt of the Keggin-type, Cs_2.5_H_0.5_PW_12_O_40_, more separable from highly polar reaction media by entrapping it into a silica matrix. Although insoluble, bulk Cs_2.5_H_0.5_PW_12_O_40_, which is characterized by very fine crystal particles (average diameter ca. 10 nm), had the disadvantage to form a colloidal solution in water or alcohol, inseparable by filtration [6]. The preparative method for POM inclusion involved the hydrolysis of TEOS in the presence of Cs_2.5_H_0.5_PW_12_O_40_ dispersed in ethanol. No additional acid catalyst was added to the reaction mixture since Cs_2.5_H_0.5_PW_12_O_40_, by its own acidity, was able to promote the hydrolysis and polycondensation of TEOS. The obtained hydrogel was dried under vacuum and then submitted to successive water extraction and calcination steps. The influence of different parameters on the success of the encapsulation was studied in detail. Notably, it was found that a high silica content was necessary to reduce the leaching of the Cs salt and that a silica/POMs ratio of 8 led to a material which could be used as a catalyst for the hydrolysis of ethyl acetate, recovered, and reused for four cycles without deactivation. This method was successfully extended to the inclusion of the corresponding free acid, H_3_PW_12_O_40_, using similar synthetic conditions (the order of the extraction-calcination steps was inverted). Both silica-included heteropoly compounds were porous materials with relatively large surface area (300–550 m^2^g^-1^) presenting meso- (Cs_2.5_H_0.5_PW_12_O_40_) and micropores (H_3_PW_12_O_40_), the difference observed in the pore diameter coming likely from the molecular size of the heteropolyanion (10 nm *versus* 1 nm for respectively Cs_2.5_H_0.5_PW_12_O_40_ and H_3_PW_12_O_40_). Inclusion of other POMs such as H_3_PMo_12_O_40,_ H_4_SiW_12_O_40_ and H_3_PW_6_Mo_6_O_40_ was reported to fail, these acids being leached from the silica matrix during the inclusion process but no explanation was put forward [7]. The scope of the silica-included H_3_PW_12_O_40_ in other acid-catalyzed reactions was further studied by Izumi *et al.* [8]. In general, the silica composite exhibited higher thermal stability than acidic ion-exchange resins and was found to be an efficient acid catalyst for liquid phase esterification and isobutene hydration with turnover frequencies larger than those of Amberlyst-15 and H-ZSM-5. Shape selectivity was also showed in the acid-catalyzed alkylation of phenol with formaldehyde giving a higher yield of the less hindered 4,4’-dihydroxydiphenylmethane isomer [8]. In 2002, Kukovecz *et al.*, as a continuation of the pioneering work of Izumi, undertook a comprehensive study by examining the effect of H_3_PW_12_O_40_ loading on the acidity of both sol-gel encapsulated and impregnated materials using pyridine adsorption, NH_3_-TPD (Temperature Programmed Desorption) and isomerization of 1-butene as a model reaction [9]. Although the acidic properties of the impregnated catalysts were superior to their sol–gel analogues, the latter exhibited higher stability. In particular, in 1-butene isomerization, the catalytic activity of encapsulated H_3_PW_12_O_40_, albeit initially lower than that of the supported materials, did not decrease significantly after the catalyst had been washed in water–alcohol solution whereas the activity of the impregnated solids dropped dramatically. This phenomenon was attributed to the dissolution of the POM in these polar solvents, as evidenced by the absence of the Keggin anion in the infrared spectra of the used catalyst. Microporous polyoxometalate based silica materials containing various amounts of H_3_PW_12_O_40_ and H_4_SiW_12_O_40_ were tested as photocatalysts in the degradation of aqueous organochlorine pesticides [10]. The materials were prepared following the synthetic and improved methods described by Izumi [6,8]. By irradiating the catalytic system with energy in the near-UV region, trace aqueous organocholorine pesticides were totally degraded and mineralized into CO_2_ and HCl under mild conditions. The photocatalytic activities of POMs were greatly improved when the POMs were entrapped by the silica matrix. However, in some cases leaching was observed during the course of the irradiation as evidenced by the appearance of a blue colour of the suspension coming from the reduced form of PW_12_O_40_^3-^ (also named heteropolyblue). As in the Izumi report [6], the authors obtained better results using a high weight ratio silica/POM (18.2 for H_3_PW_12_O_40_ and 32.2 for H_4_SiW_12_O_40_) to ensure a better inclusion and suppress the release of the Keggin anion yielding catalytic systems which could be reused 8 times without losing their initial activity. The authors commented on the integrity of the Keggin anion and the interactions between the POM and the silica network based on their DR-UV and FT-IR spectroscopic results. They stated that infrared absorbances characteristic of POMs in the hybrid, compared to the parent POMs, indicated the primary structure of the POM has been retained and that a strong chemical interaction exists between the POM and the silica surface. In the acidic synthesis conditions the silanol groups would be protonated and act as a counterion for the polyanion leading to the generation of species such as (≡Si-OH_2_^+^)(H_2_PW_12_O_40_^-^) or (≡Si-OH_2_^+^)(H_3_SiW_12_O_40_^-^). Thus, the reaction of the polyanion with SiO_2_ may be better described as a true acid-base reaction between the silanol groups (acting as the base) and the polyanion (acting as a Brønsted acid), accounting for the stabilization effect of the silica framework, rather than a simple ion exchange reaction. Similar chemical interactions were reported to occur during the formation of microporous decatungstates between M_4_W_10_O_32_ (M = Na^+^, nBu_4_N^+^) molecules and the silica network yielding (≡Si-OH_2_^+^)(M_3_W_10_O_32_^-^) surface species [11]. On the other hand, Thouvenot [12] and Lefebvre [13], respectively, for H_4_SiMo_12_O_40_ and H_3_PW_12_O_40_ supported on silica via impregnation methods, also proved such interactions through ^29^Si- and ^31^P MAS-NMR experiments.

In general, the encapsulation of POM clusters inside the silica framework leads to hybrid composites which exhibit higher performances over their supported counterparts in terms of activity, selectivity but also recycling efficiency [6,10,11]. More importantly, leaching of the immobilized POM clusters has been reduced, if never completely suppressed, during the catalytic processes. Owing to their molecular and electronic structural diversity and for practical applications, and indeed to the simplicity of their preparation, studies dealing with the inclusion of polyoxometalates into silica matrix continue to appear regularly [14,15]. Among many applications one can cite the environmentally friendly photo-degradation of organophosphorus pesticides by encapsulated decatungstates [11], the gasochromic sensing activity of hybrid silica material containing phosphomolybdic acid for H_2_S and SO_2_ gases at room temperature [16], the luminescent properties of materials containing heteropolyoxometalates of the type K_13_[Eu(SiMo_x_W_11-x_O_39_)_2_] incorporated into organically modified xerogel matrices [17] and the catalytic methoxylation of α-pinene catalyzed by molybdophosphoric and tungstosilicic acids immobilized on silica [15].

However, due to the relatively small size of their pores (<2 nm), these hybrid materials may suffer from similar diffusional problems as zeolites for applications involving larger substrates. Therefore, to extend the scope of POM heterogeneous catalysis, the development of new routes to solid POMs with larger pore sizes and higher surface areas is of primary importance.

## 3. Mesostructured Silica Based Materials

A new class of mesoporous silicates and aluminosilicates, called M41S, has been developed by a research group at Mobil Oil by combining the sol-gel processing with supramolecular templating agents [18,19,20,21]. These silica-based materials, structured at the mesoscopic level, were synthesized by introducing in the hydrothermal sol-gel synthesis entities able to self-assemble (surfactant) (Figure 1, path B). The existence of an attractive interaction between the template and the silica precursor is necessary to ensure inclusion of the structure directing agent (SDA) without phase separation. These materials are characterized by very large specific surface areas and tunable ordered pore systems with narrow pore size distributions. Surfactant-templated ordered mesostructured materials like MCM-41, MCM-48, and SBA-15 are known to be excellent supports for the dispersion of POMs. However, leaching of POMs from the surface or migration to aggregate on the outer surface of the support as larger clusters usually occurred when water was formed as a side-product in the reaction (*i.e.,* esterification reactions) or in applications using polar solvents. Since no strong linkage exists between the POMs and the silica surface via the impregnation route, the widespread use of the modified hybrid materials remains still limited for industrial applications. To avoid these problems, new synthetic routes have been reported for the immobilization of polyoxometalates by stronger chemical bonding through organic modification of mesoporous silica. For example, H_6_PMo_9_V_3_O_40_ was immobilized inside the channels of MCM-41 which had been modified by amine functional groups: in this case the POM was held through an ionic bond (anion, ammonium) [22]. Transition-metal substituted polyoxometalates of the type [M^II^(H_2_O)PW_11_O_39_]^5-^ (M = Co, Zn), have also been chemically anchored to aminopropyl modified silica surfaces through dative bonding [23].

Another approach to stabilize the POM into the silica host matrix is to encapsulate it during the formation of the ordered silica mesostructure. This promising route has been recently described by different authors to yield well-dispersed and stabilized POM clusters containing nanocomposites [24,25,26,27,28,29,30,31,32,34,35,36,37]. The incorporation of bulky POM clusters during the gel formation may lead to substantial modifications of the resulting supramolecular assemblies giving rise to more disordered hybrid solids. Recall that the mild conditions used in sol-gel chemistry afford systems mostly under kinetic control and that slight changes of experimental parameters may have a profound influence on the morphology, structure and properties of the resulting solids.

Here, we will discuss the variations of the synthetic procedures to encapsulate POMs into organized mesoporous silicas and discuss the key factors which are necessary to take care of to preserve structural integrity of the POM and textural properties of the support upon site inclusion. In Table 1, we have summarized the principle experimental conditions and characteristics of the obtained materials reported to date. Although the synthesis of ordered mesoporous silica materials can be achieved over a wide range of experimental conditions, the inclusion of POMs limits the possibilities. Notably, one cannot use syntheses in alkaline medium and/or involving cationic alkylammonium halide templates because POMs are usually unstable at basic pH and the ammonium POM salt is insoluble in water (leading to precipitation and hence poor dispersion of the active phase).

**Table 1:** Major synthetic routes to mesostructured silica based materials containing entrapped POMs.

Polyoxo-metalate	Other function	Structure directing agent	Type of mesostructured silica	Type of bonding	Template removal technique	Refer-ence
H_3_PW_12_O_40_		CTAB + Triton (TX-100)	MSU	Electrostatic bond	Calcination	[24,25,26]
H_3_PW_12_O_40_	H_2_PtCl_6_^.^6H_2_O^(a)^	CTAB + Triton (TX-100)	MSU	Electrostatic bond	Calcination	[27]
H_3_PW_12_O_40_		MTMABr	SBA-3	Electrostatic bond	Calcination	[28]
H_3_PW_12_O_40_		Pluronic 123	SBA-15	Electrostatic bond	Calcination	[29,30,32]
H_3_PW_12_O_40_ H_3_PMo_12_O_40_		CTAB + Pluronic 123	SBA-15	Electrostatic bond	Subsequent calcination & extraction	[31]
H_3_PW_12_O_40_	[Pt(NH_3_)_4_]Cl_2_^(b)^	Pluronic 123	SBA-15	Electrostatic bond	Calcination	[34]
Na_2_HPO_4_ + Na_2_WO_4_		Pluronic 123	SBA-15	Electrostatic bond	Extraction	[35]
K_8_SiW_11_O_39_ (R_4_N)_8_SiW_11_O_39_		Pluronic 123	SBA-15	Covalent bond	Extraction	[37]

^(a)^ Introduced simultaneously with H_3_PW_12_O_40_. ^(b)^ Introduced post-synthetically by impregnation.

### 3.1. MSU-type Mesoporous Material

In several reports, Toufaily *et al.* [24,25,26] studied the direct incorporation of Keggin-type H_3_PW_12_O_40_ into organized silica molecular sieves based on the association of two surfactants: a non-ionic one (S^0^), polyethyleneglycol-4-*tert*-octylphenylether (Triton X-100), and an ionic one (S^+^), cetyltrimethylammonium bromide (CTAB). The effects of several synthetic parameters on the structure of the mesoporous solid were examined (pH of the blend, POM loading, molar ratio S^0^/ S^+^). Stabilized materials with high loading and intact Keggin anion, albeit exhibiting poor long range ordering, were obtained by restricting the pH domain to 4–6 and by using an additional cationic surfactant together with non-ionic Triton X-100. Extraction performed on incorporated and impregnated materials showed that H_3_PW_12_O_40_ was still maintained in the solid prepared by direct synthesis whereas complete leaching was noticed for impregnated solid [25]. The authors attributed the stabilization effect of silica to interactions of the type (≡Si)_m_^+^(H_3-m_PW_12_O_40_)^m-^ as shown below:

H_3_PW_12_O_40_ + m(≡Si-OH) ➔ (≡Si)_m_^+^(H_3-m_PW_12_O_40_)^m-^ + H_2_O (with m = 1–3)

Further insights concerning the location of POM entities in the hybrid solid were obtained from ^31^P MAS-NMR studies leading to an estimation of the mobility of the POM clusters (via the measurement of spin–lattice relaxation times, *T_1_*) [24]. The samples prepared by direct incorporation exhibited a *T_1_* 30 times higher than that of the species prepared by impregnation which suggests a weaker mobility of the POM units and demonstrates that they are likely located in the walls.

The role of the cationic surfactant was investigated in the synthesis of functionalized MSU-type mesoporous silica which was carried out in an acidic medium in the presence of H_3_PW_12_O_40_ [26]. The ordering of the inorganic lattice was mediated by either a mixture of non-ionic (S^0^) and cationic (S^+^) surfactants (TX100/CTAB) or by only a non-ionic one (Tween 60 and Tergitol 15-S-12). Fluorine anions were used as catalysts to accelerate the polycondensation of silica and improve structural ordering of the material. In general, the calcined POM materials exhibited poor mesoscopic order with no long-range periodicity likely due to the wormhole structure of MSU type solids. Where the non-ionic surfactants were employed, the authors attributed the inclusion of H_3_PW_12_O_40_ to the acidic protonation of the ethoxy groups of the polyoxyethylene alkyl ether surfactant whereas for mixed micellar systems, the incorporation has been ascribed to interactions with the cationic co-surfactant. Increasing the electrostatic interactions (increasing S^+^ concentration) gave rise to a more highly organized material.

Based on this work, Hamad *et al.* prepared dual functionalized materials via direct incorporation of H_3_PW_12_O_40_ and H_2_PtCl_6_·6H_2_O at various ratios into MSU-type materials using the same CTAB/TX100 mixed micellar system [27]. The presence of different POM local structures was evidenced by the presence of several peaks in solid-state ^31^P-NMR. They observed not only H_3_PW_12_O_40,_ which may or may not be involved in a weak interaction with the silica surface, but also mixed Pt/POM complexes of the type [PW_11_O_39_Pt] or [PW_11_O_39_Pt–O–PtPW_11_O_39_] which would arise from the reaction between Pt and lacunary (PW_11_O_39_)^7−^. The catalytic activity of the calcined Pt/POM silica mesoporous solids for NO*x* reduction showed that optimal activity (up to 97%) was obtained with a catalytic system characterized by low Pt loading (0.5 wt %) and a moderate amount of H_3_PW_12_O_40_ (~ 15%), and in the temperature range 225–250 °C and for a large oxygen concentration (0.5–10 vol %). Under identical reaction conditions, only moderate conversion was achieved for the monofunctionalized solids H_3_PW_12_O_40_/MSU and Pt/MSU.

### 3.2. SBA-3-Type Mesoporous Materials

In 2003, Nowińska *et al.* described the synthesis of SBA-3 type silica by direct incorporation of H_3_PW_12_O_40_ and H_5_PMo_10_V_2_O_40_ POM clusters under acidic pH and in the presence of meristyltrimethylammoniun bromide (MTMABr) as unique structure directing agent [28]. Compared to plain SBA-3, a slight excess of MTMABr was introduced in the gel to prevent the neutralization of all template by H_3_PW_12_O_40_ or H_5_PMo_10_V_2_O_40_. Modified solids with proper mesostructures could be obtained with up to 20 wt % of POM in the system, using calcination as the template removal method. Accessibility and acidity of the POM sites were confirmed by their activity for cumene cracking reaction which was similar to that reported for impregnated analogue solids. The authors reported on the stability, or rather the instability, of the materials with respect to alcohols (leaching again being a major problem) and drew interesting conclusions about the localization of POM inclusion based on the comparison of the stability of their hybrid materials with that of simply impregnated mesoporous silicas. Leaching of the POM by polar molecules (alcohol, water) was complete in all cases, but, significantly, in the case of the authors’ hybrid material, this leaching led to the collapse of the mesostructure. In the case of impregnated materials, the silica structure was unaffected. Clearly, the POM plays a role in the structural integrity of Nowińska’s hybrid, and the authors suggest that this indicates that the Keggin units in these materials were mainly located in the walls.

### 3.3. SBA-15-Type Mesoporous Materials

The loss of mesoporous ordering and poor stability of SBA-3 type materials could stem from the thin wall thickness intrinsic to the nature of the template and assembly pathway. On the other hand, SBA-15 mesoporous silica is well-suited as a support for immobilizing functional groups. Its large, uniform pore diameter (~ 6 nm) provides ample room for reactant and product diffusion, and its thick walls (50–60 Å for SBA-15 *versus* 15 Å for SBA-3 silica) provide hydrothermal stability. In addition, the acidic conditions of the synthesis should be a key factor to maintain the integrity of the primary structure of the included POMs. Many reports have recently appeared of SBA-15 synthesis protocols to incorporate POM clusters into the silica framework. These studies vary both in terms of the nature of the POM introduced, the order of the introduction of the different components (template/POM/TEOS) and the nature of the interactions (covalent or electrostatic) between POM and the inorganic network. All these aspects will be illustrated by a few examples. The preparation of mesoporous SBA-15 type silica containing 12-phosphotungstic acid has been reported by different groups [29−32]. The basic synthetic procedure involves the hydrolysis and condensation of TEOS in acidic medium in the presence of H_3_PW_12_O_40_ and a non ionic triblock copolymer as the template, Pluronic 123 (EO_20_PO_70_EO_20_ with EO, ethylene oxide and PO, propylene oxide). Some variations of the classical preparation were described leading to materials with quite different properties in term of morphologies, structural and textural order, loading and hydrothermal stability. For example, some have mentioned a prehydrolysis of TEOS (the length of time varying between 30 min to 20 hours) in the presence of Pluronic 123 before adding H_3_PW_12_O_40_ as a key synthetic variation [29,31], while others proceeded by simultaneous hydrolysis and condensation of TEOS with H_3_PW_12_O_40_ in the presence of the template [32]. Dufaud and co-workers [31] also used a mixture of structure directing agents (CTAB and Pluronic 123), the idea being that mixed micelles with ionic surfactants is an easy way to change the surface charge distribution (at the surface of the micelle) leading to stronger interactions between the template, inorganic precursor and included POM. Indeed, stabilized materials with intact Keggin units were obtained with a SDA mixture Pluronic/CTAB molar ratio of 1 to 0.8 whereas in the absence of CTAB or with a higher CTAB concentration (1:1.6 molar Pluronic/CTAB ratio) the primary Keggin unit was respectively partially or completely destroyed as evidenced by ^31^P-NMR spectroscopy [31].

In all the syntheses, a hydrothermal treatment of the gel was performed after all of the components have been introduced to allow the polymerization to occur (ageing step), usually at temperatures ranging from 80 to 110 °C. After drying the resulting solid, calcination in air was usually employed to remove the Pluronic 123 [29,30]. To reinforce the interactions between POM clusters and the silica matrix and hence trap more firmly the embedded POM in the solid, some authors introduced a calcination step in the synthesis protocol [31,32]. Guo *et al.* carried out the calcination of the dried gel particulates successively at 80, 100 and 120 °C under vacuum before removing the template from the pores either by ethanol extraction or by calcination at 420 °C. In both cases, high H_3_PW_12_O_40_ loadings were obtained indicating that the “calcination” step which can be considered here more as a thermal treatment (no surfactant was removed at this stage) inhibited the loss of the Keggin unit during template extraction process. The authors confirmed the integrity of the Keggin unit by Raman scattering studies excepting for the case where calcination was performed at high temperatures (up to 520 °C) [32]. On the contrary, in the protocol described by Dufaud *et al.*, the calcination in air at 500 °C overnight was accompanied by quantitative removal of the P123 template [31]. Although high POM loadings remained in the material the primary Keggin unit was partially destroyed upon this treatment as evidenced by solid state ^31^P-NMR. However, structurally intact and accessible POM moieties could be obtained after a subsequent extraction of the calcined material with methanol. The authors hypothesized that calcination could lead to some restructuring of the surface by reducing the size of the entrance of the micropore host which in turn would inhibit extraction. The methanol-mediated reformation of the Keggin structure could then take place in the solid.

Some studies seem to indicate that POM clusters in the hybrid materials are likely located into the silica framework rather than held at the surface of the pores. For example, Yang *et al.* when comparing the mesostructure and acidic properties between sol–gel derived and impregnated samples showed that the sol-gel material exhibited a better stability in systems involving polar solvent and that the changes in textural properties by POM introduction (decrease in surface area, total pore volume and pore diameter) were smaller than those observed for impregnated sample [30]. They suggested that this indicates the presence of a considerable amount of POM in the pore walls of the sol-gel material. One could also cite Yun *et al.* who, based on nitrogen adsorption-desorption measurements, proposed that the loaded POM did not exist in the inner part of the mesopores but rather in the micropores of the SiO_2_ framework [29]. This is not surprising given that the sol solution was aged at 80 °C which is known to produce micropores [33].

**Figure 2:**
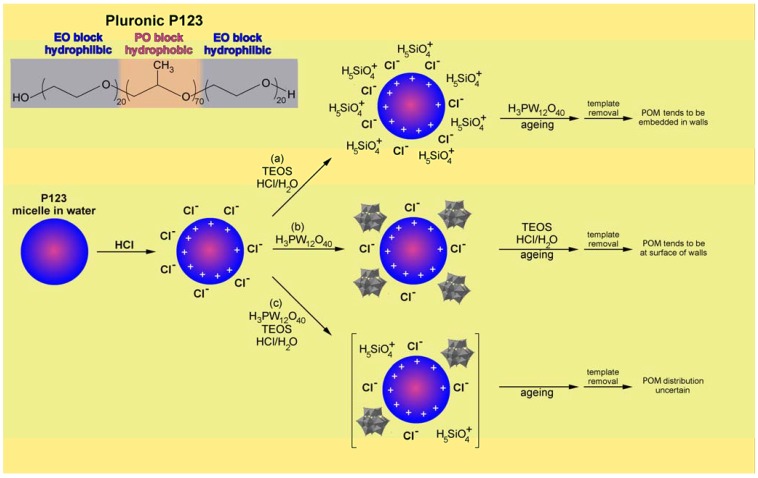
Proposed synthesis mechanism for POMs encapsulation into the silica framework of SBA-15 type silica: (a) Prehydrolysis of TEOS in the presence of P123 followed by introduction of H_3_PM_12_O_40_ [29,31] (b) Introduction of H_3_PM_12_O_40_ in the P123 system followed by TEOS addition [34] (c) Simultaneous introduction of TEOS and H_3_PM_12_O_40_ in the P123 structure directing agent solution [32].

The localisation of the POMs in the silica framework can be explained in terms of the commonly accepted mechanism of solid assembly. As proposed above for non-structured sol-gel based materials [10,11], the acidic conditions of the synthesis resulted in the presence of ionic species in solution. The ethylene oxide (EO) moieties of the surfactant, Pluronic 123 (EO_20_PO_70_EO_20_) could be associated with hydronium ions as PO_m_[(EO).H_3_O^+^)]_x_….xCl^-^ which could further react with monomers like (SiO_4_H_5_)^+^ originating from the hydrolysis and protonation of TEOS (Figure 2a).

The POM clusters dissolved in water are thus easily and homogeneously incorporated in the hydrophilic/silicate chain interface *via* electrostatic interactions or hydrogen bonding, subsequently affording well dispersed POM clusters throughout the solid upon solidification of the silica network. In the case of the synthesis described by Dufaud *et al.* this organized assembly pathway would be reinforced by the presence of an additional cationic surfactant (cetyltrimethylammonium bromide) which could directly interact with some of the H_3_PW_12_O_40_ to form the corresponding ammonium salt, hence forcing the POM to remain in the polar medium and consequently in the walls of the material [31]. To attempt better dispersion of POM clusters into the SBA-15 silica network, Gagea *et al.* reported a direct synthesis which involved the introduction of H_3_PW_12_O_40_ in an acidified solution of P123 triblock copolymer, the SBA-15 mesostructuring agent [34]. In this approach, H_3_PW_12_O_40_ was introduced before the hydrolysis of TEOS. Based on the supramolecular mechanism of SBA-15 formation denoted S^0^H^+^X^-^I^+^, with S^0^, H^+^, X^-^ and I^+^ referring to as respectively Pluronic 123, hydronium cation, halogen anions and positively charged silica source [20], the authors proposed the following pathway to account for the synthesis mechanism in the presence of H_3_PW_12_O_40_ (Figure 2b) [34]. As previously described, the P123 micelles would be protonated through the ethoxy groups, the positive charge being compensated by the chloride anion. The weak conjugate base of H_3_PW_12_O_40_ could then substitute the Cl^-^ anions leading to strongly attached and molecularly dispersed Keggin units on the P123 micelle before the addition of the silica source. This exchange is likely given the 24 hours allowed at this step in the synthesis. TEOS as (SiO_4_H_5_)^+^ cations would then interact with the anionic shell surrounding the P123 micelles. The subsequent hydrothermal treatment which creates the silica network would thus trap both Cl^-^ and heteropoly anions between P123 and the silica.

SBA-15 ordered mesoporous silica materials containing up to 40 wt % stabilized H_3_PW_12_O_40_ were synthesized via this route and converted into bifunctional H_3_PW_12_O_40_/Pt catalysts presenting acid and hydrogenation–dehydrogenation functions. The performances of these materials were investigated in the conversion of n-decane under different catalytic conditions [34].

**Figure 3:**
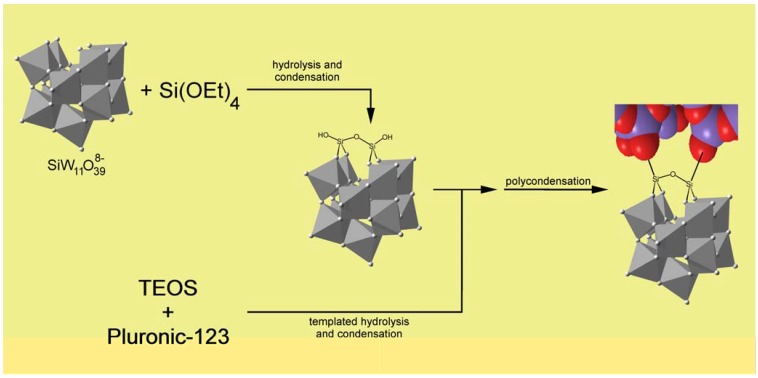
Inclusion of lacunary SiW_11_O_39_^8-^ and covalent bonding with the framework.

Another route to incorporate POMs into the SBA-15 silica framework has been recently reported by Shi *et al.* [35]. This approach relied on the *in situ* formation of the Keggin unit H_3_PW_12_O_40_ by directly introducing Na_2_HPO_4_ and Na_2_WO_4_ as P and W sources respectively into the initial sol-gel system during hydrolysis of TEOS. SBA-15 mesoporous solids with highly ordered hexagonal mesostructures were obtained for POM contents varying between 13.3–20.7 wt % suggesting that the *in situ* formation of H_3_PW_12_O_40_ did not disturb the assembly pathway. The Keggin units retained their integrity as confirmed by FTIR and UV-Vis spectroscopy and were quite stable as evidenced by the high amount of POM remaining after repetitive ethanol and water washings employed to extract the Pluronic 123. These hybrid materials were used successfully in acid-catalyzed cracking reactions with small (cumene) and bulky (1,3,5-triisopropylbenzene) molecules. The catalyst maintained high activity in esterification of acetic acid with ethanol over five reuse cycles whereas activity dropped rapidly for analogous silica supported POMs prepared by post-synthetic impregnation [35]. The authors attributed the increased stability of encapsulated POMs to the existence of stronger interactions with the silica surface than those operant in impregnated samples. The scope of these hybrid materials was further investigated by Wang *et al.* in the preparation of 1,1-diacetates from aldehydes under mild reaction conditions [36]. The catalysts showed high activity and selectivity and were efficiently reused without significant loss of activity. Recently, polyoxometalate compounds were introduced onto periodic ordered mesoporous silica by covalent linkages [37]. This interesting approach involves the co-condensation of TEOS with Keggin-type monovacant polyoxometalate of the type SiW_11_O_39_^8-^ in the presence of block copolymer P123. A similar approach was described in 2002 by Guo *et al.* for the preparation of macroporous structured silica based POM materials (*vide infra*) [38]. Monovacant tungstosilicate SiW_11_O_39_^8-^, which is an lacunary Keggin (SiW_12_O_40_^4-^) fragment, was reported to react with silicon atoms of organotrialkoxysilane by a nucleophilic mechanism under acidic conditions to yield organo-species of the type SiW_11_O_39_[O(SiR)_2_]^4-^ [39]. In this reaction, two tetrahedral Si atoms were inserted into the vacancy of SiW_11_ via Si–O–W bonds, with the concomitant formation of a Si–O–Si bridge between the two Si atoms, leading to the saturation of the lacunary Keggin structure. Based on this synthesis, Zhang *et al.* investigated the reactivity of SiW_11_O_39_^8-^ with TEOS, traditional source of silica in the preparation of silica based mesoporous materials, to produce molecule such as SiW_11_O_39_[O(SiOH)_2_]^4-^ which then could act as an intermediate by which the condensation between POM and silica species could occur. Tetraalkylammoniun and potassium salts of SiW_11_O_39_[O(SiOH)_2_]^4-^ species were isolated and fully characterized and used as a reference to confirm the *in situ* generation of the latter from SiW_11_O_39_^8-^ and TEOS during the formation of the mesostructured silica framework. The synthetic scheme of the incorporation and bonding of SiW_11_O_39_^8-^ with the silica framework is depicted below (Figure 3). The authors proposed that the POM species are bound on the surface instead of being encapsulated into the SiO_2_ network of the walls even though some of them may have been entrapped into the small pores of the walls [37].

The investigation of the synthesis conditions showed that the prehydrolysis time of TEOS was a key factor to obtain materials with highly ordered mesostructure and intact Keggin units immobilized in channels by covalent linkages with the mesopore walls. The hybrid materials exhibited also higher stability in water-leaching experiments compared to impregnated samples suggesting that the nature of the link but also the strategy of synthesizing the material was important.

## 4. Macrostructured Silica Based Materials

Ordered macroporous materials in the submicronic range (∅_pore_ > 50 nm) are of particular importance due to their potential applications as adsorbents, battery materials, three-dimensional photonic crystals with optical band gaps and catalyst supports [40,41]. In the field of catalysis, organized macroporous arrays should provide good accessibility to reactive site owing to their improved transport properties and optimal diffusion. Three-dimensional ordered macroporous (3DOM) solids, also referred to as inverse opals, with narrow macropore size distributions and high porosity are generally obtained by colloidal crystal templating (Figure 1, path C). This approach is based on the use of colloidal crystals (usually colloidal suspension of polystyrene spheres (PS)) as template. The interstitial space between the close-packed PS spheres is then impregnated with an alkoxide precursor which is capable of solidification followed by elimination of the organic template by heating or washing. The three dimensional porous network is thus liberated.

**Figure 4:**
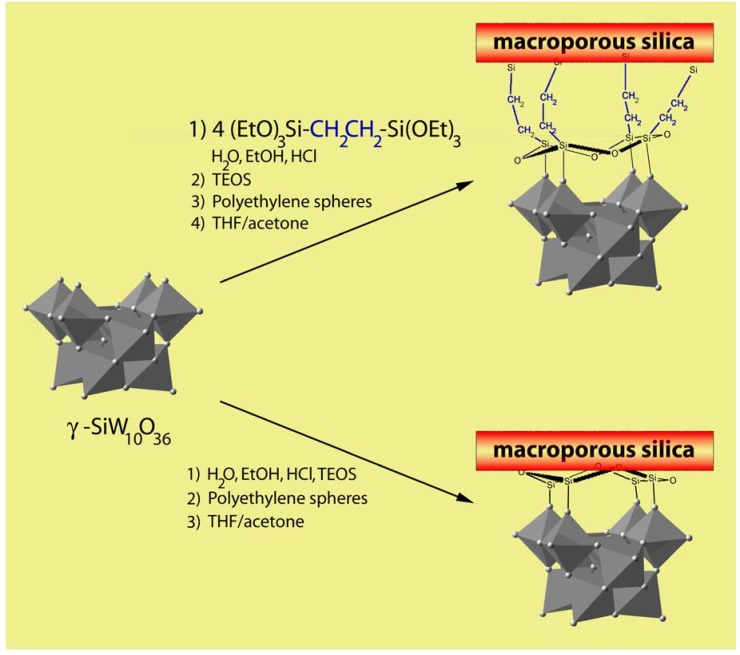
3DOM silica functionalized with γ-tungstosilicate clusters of the type [γ-(RSiO)_4_SiW_10_O_36_]^4-^.

Based on this approach, polyoxometalates functionalized 3DOM silicates have been recently developed by different groups [42,38]. Using a direct synthesis procedure, Schroden *et al.* reported the incorporation of divacant tungstosilicate cluster of the type [γ-SiW_10_O_36_]^8-^ into the wall structures of macroporous silica through covalent bonding [42]. In this study, the authors utilized the reactivity of lacunary polyoxometalates toward siloxanes previously described by Mayer and co-workers [43,44] to create a covalent bond, W-O-Si, between the POM and the silica precursor, either in the presence or the absence of bifunctional organosiloxane linkers (Figure 4). Further condensation of the silicate framework around polystyrene colloidal crystals led to the incorporation of the POM containing species to the 3DOM [42].

Template removal by extraction was found to be a key parameter to the obtention of stable hybrid macroporous materials with structurally intact POM units homogeneously distributed throughout the material. In contrast, calcination procedures led to the decomposition of POMs and aggregation of tungsten oxide species on the outer-surface of the solid. The different modes of POM linking to the silica matrix depended on the synthetic methods employed (through an organic spacer or directly attached), as confirmed by single-pulse and CP ^29^Si MAS-NMR technique. Evaluation of the POM-functionalized silica materials in the epoxidation of cyclooctene using hydrogen peroxide as co-oxidant showed high selectivity toward the epoxide product. POM clusters linked to the silica framework by bis(silyl)ethane groups remained intact after catalytic testing whereas those directly condensed with the silica matrix, while exhibiting higher activity, underwent partial degradation. The authors draw a parallel between this behaviour and previously observed correlations between POM decomposition by hydrogen peroxide and catalytic epoxidation efficiency [42].

An extended work with monovacant lacunary Keggin-type polyoxometalates [XW_11_O_39_]^8-^ (with X = P^5+^, Si^4+^, Ge^4+^, B^3+^) was performed successively by Guo *et al.* to prepare composites capable of photocatalytic activity [38]. Like Shroden and co-workers [42], they took advantage of the specificity of monovacant lacunary POMs to react via their nucleophilic surface oxygen atoms with TEOS to covalently anchor the POM cluster to the silica matrix. Permeating the obtained hybrid [XW_11_O_39_]^8-^-SiO_2_ sol into the voids of PS spheres yielded three-dimensionally ordered macroporous hybrid silica materials with pore diameters ranging from 285 to 385 nm. Adaptation of the reaction conditions (solubility of the inorganic [XW_11_O_39_]^8-^ species in water, control of the acidification of the sol mixture, choice of the method for template removal) was necessary to achieve materials with molecularly well dispersed [XW_11_O_39_]^8-^ units. Based on different spectroscopic techniques (FTIR, ^31^P NMR) the authors confirmed that the primary Keggin structures remained intact regardless of the functionality of the polyanions, and that the surface of the parent [XW_11_O_39_]^8-^ units achieved saturation through chemical grafting of silanol groups from the silica network. The photocatalytic degradation of aqueous malic acid used as benchmark reaction showed that [XW_11_O_39_]^8-^ sites retained their photocatalytic activity in the hybrid materials without observable leaching of POM clusters from the used catalyst. The authors attributed the improved stability of these materials over analogs prepared by conventional impregnation technique to the strong covalent interactions between silanol groups and the lacunary POM molecules in the composites.

## 5. Other Oxides

Farhadi *et al.* described the preparation of polyoxometalate–zirconia (POM/ZrO_2_) nanocomposite through entrapment of H_3_PW_12_O_40_ into zirconia matrix by sol–gel technique [45]. The procedure involves the hydrolysis of zirconium (IV) *n*-butoxide, Zr(n-OBu)_4_, as the ZrO_2_ source in the presence of phosphotungstic acid under acidic conditions. The resultant materials contained 20 wt % of intact POM units which were firmly maintained in the material via electrostatic interactions and hydrogen bonding with the ≡Zr–OH groups of the ZrO_2_ support. In acidic medium, these latter are protonated to form ≡Zr–OH_2_^+^ species which act as the counter ion of polyanion, and yield (≡Zr-OH_2_)^+^(H_2_PW_12_O_40_)^-^ species by acid-base reactions, similar to what was observed in the case of silica. This strong chemical interaction between the polyanion and the zirconia surface was confirmed by red shifts of characteristic bands of POM in the POM/ZrO_2_ nanocomposite relative to the pure POM in the FTIR and UV-Vis spectra. Hybrid materials with specific area up to 300 m^2^g^-1^ and particles h²aving a narrow size distribution in the range of 10–15 nm were obtained making the POM/ZrO_2_ nanocomposite promising as an environmentally friendly heterogeneous catalyst for photocatalytic transformations. The performances of POM/ZrO_2_ were evaluated in the selective aerobic oxidation of alcohols into corresponding carbonyl compounds under mild conditions (oxygen atmospheric pressure, room temperature). Various primary and secondary benzylic alcohols were efficiently oxidized into the corresponding aldehydes and ketones in the presence of photoexcited POM/ZrO_2_ nanocomposite under O_2_ atmosphere. When the reaction was carried out under nitrogen, the desired product was obtained in low yield (< 6%), stressing the important role that O_2_ plays in the reoxidation of the reduced POM. The scope of the reaction was also extended to nonactivated aliphatic alcohols for which longer reaction times were necessary to achieve good yields and to heteroaromatic alcohols which were converted to the corresponding aldehydes in high yields without oxidation of the heteroatoms (*i.e.,* N, S). The difference in reactivity observed between benzylic and aliphatic alcohols allowed the authors to perform selective oxidation of *vicinal*-diols containing benzylic and non-benzylic hydroxy groups by choosing an appropriate reaction time. Indeed, α-ketols were thus cleanly obtained at short reaction times via selective oxidation of the hydroxyl group α to the benzene ring. In addition, recycling of the POM/ZrO_2_ photocatalyst showed no significant loss of activity and selectivity upon several cycles.

**Figure 5:**
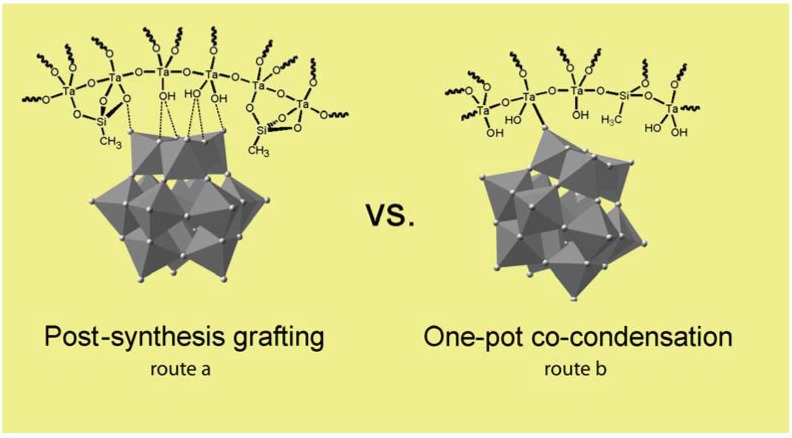
3D interconnected mesoporous organic–inorganic hybrid catalysts prepared *via* dual functionalization through (a) post-synthesis grafting (b) one-pot co-condensation route.

In 2008, Xu *et al.* reported the preparation of mesoporous polyoxometalate-tantalum pentoxide composite, H_3_PW_12_O_40_/Ta_2_O_5_, using a sol–gel hydrothermal route in the presence of Pluronic 123 [46]. TaCl_5_, precursor of the Ta_2_O_5_ support, was first introduced on a P123 alcoholic solution followed by addition of H_3_PW_12_O_40_. The subsequent hydrothermal treatment of the sol and template removal by extraction with boiling ethanol led to 3D interconnected mesoporous hybrid composite with high surface area (130 m^2^ g^-1^) containing homogeneously dispersed Keggin units throughout the solid, as evidenced by wide-angle XRD. Various spectroscopic methods (FTIR, Raman, ^31^P-NMR and XPS) were used to get better insights into the structural nature of the POM and the type of interactions between this latter and Ta_2_O_5_ support. Taken together, the data suggest that the integrity of the primary Keggin unit was retained after incorporation into the material and that strong interactions between the Keggin unit and Ta_2_O_5_ framework exist in the composite. Based on the similarities observed between Ta^5+^ and W^6+^ in term of electronegativity and ionic radius, the authors tentatively proposed that the terminal W=O moieties within H_3_PW_12_O_40_ coordinate the surface Ta-OH groups through W-O-Ta bonds leading to the formation of (TaOH_2_)_n_^+^[H_3-n_PW_12_O_40_]^n-^ species on the surface. The H_3_PW_12_O_40_/Ta_2_O_5_ composite exhibited higher activity, selectivity and stability than either molecular H_3_PW_12_O_40_ or Ta_2_O_5_ support for the acid-catalyzed esterification and transesterification for biodiesel production. This was attributed to the presence of stronger acidic sites originating from the strong interactions which exist in the solid between the Keggin unit and the hydroxyl groups of the tantalum pentoxide support which could result in lower proton releasing energy and hence increased Brønsted acidity of the material [46]. However, some limitations affecting the performances of the catalyst remained. In esterification reaction, the water by-product was not efficiently removed from the surface owing to the hydrophilicity of the H_3_PW_12_O_40_/Ta_2_O_5_ composite, preventing the approach of the reactants and hence slowing down the reaction rate. Likewise, for transesterification reactions, the strong adsorption of glycerol on the H_3_PW_12_O_40_/Ta_2_O_5_ surface hindered the diffusion and adsorption of triglycerides along the channels and, in some cases, led to the deactivation of the catalyst. Therefore continuing work was performed by Xu *et al.* to enhance the hydrophobicity of H_3_PW_12_O_40_/Ta_2_O_5_ composite [47]. This was achieved by simultaneous co-condensation of organosilanes of the type RSi(OCH_3_)_3_ (R = Me, Ph) with H_3_PW_12_O_40_ and TaCl_5_ in the presence of non-ionic surfactant following the procedure described in their previous work [46]. 3D interconnected mesoporous organic–inorganic hybrid catalysts, Ta_2_O_5_/SiO_2_-[H_3_PW_12_O_40_/R] (R = Me or Ph), functionalized with both alkyl groups and POM clusters, were thus prepared and their performances evaluated towards esterification of free fatty acids and transesterification of triglycerides in soybean oil to produce fatty acid methyl esters (main components of biodiesel). A complete study regarding the influence of different synthetic parameters and reaction conditions (catalyst preparation route, relative functional groups ratios, and molar ratios of oil to methanol) on the catalytic activity and selectivity as well as on the recyclability of the hybrid materials was undertaken [47]. In general, dual functionalized hybrid catalysts exhibited much higher catalytic activity to the above reactions than their alkyl-free counterparts and could be reused at least 4 times without noticeable deactivation. The authors attributed this enhanced performance to the presence in the solid of an alkyl functional group which renders the local micro-environment around the active site more hydrophobic. The differences in term of activity and stability observed between multifunctionalized grafted and co-condensed hybrid materials arose from the preparation routes which led to materials with different properties (Figure 5). While co-condensation approach yielded a solid with homogeneous random dispersion of both alkyl groups and Keggin units throughout the solid (Figure 5, route b), the grafting method led to a less uniform distribution of the two functionalities (Figure 5, route a). Indeed, the reactive organosilanes may react preferentially with the Ta-OH groups on the outside of the particle and at the pore aperture resulting in pore clogging hence masking any benefit that surface hydrophobicity at the active site might bring. Moreover, the weak interactions between POM clusters and the surface hydroxyl groups (mainly through hydrogen bonding) in the grafting route compared to the strongly bound (TaOH_2_)_n_^+^[H_3-n_PW_12_O_40_]^n-^ species in the co-condensed route, resulted in leaching of most of the H_3_PW_12_O_40_ during catalytic experiments.

Recently a new field of research has emerged based on the direct assembly of POMs and organic cations leading to the formation of a variety of supramolecular structures which have been used in various domains [48,49,50,51,52,53,54]. Various polyoxometalates have been used although Keggin structures were preferred for their higher stability and fully symmetric structure. The organic cation was generally in the form of an ammonium group bearing one or two long alkyl chains. The resulting material can display an amorphous or a crystalline structure and among their reported applications, one can use them as templates for the polymerization of organic or inorganic monomers. Zhao *et al.* illustrated the diversity of applications possible with such systems [55]. A polyoxometalate displaying interesting photoreduction properties (EuP_5_W_30_O_110_^12-^) was reacted with a long chain organic cation, 11-hydroxylundecyldimethylammonium, leading to a surfactant-encapsulated cluster. This organic-inorganic complex possessed an inorganic core of POM and an organic shell constituted of hydrophobic surfactant with hydroxyl groups pointing at the outside of the supramolecular structure. The resulting material was soluble in a water/ethanol mixture and co-condensed with alkylorthosilicate, Si(OEt)_4_, in the presence of ammonia leading to stable silica spheres containing surfactant-encapsulated POM. The POMs were shown to be uniformly dispersed and firmly maintained in the hybrid silica spheres with intact structure and properties. Although, embedded in the hydrophobic microenvironment of the surfactant, ultraviolet light can penetrate this outer sphere and trigger the photoreduction of the POM leading to a reduced form, heteropolyblue, inside the silica sphere. The heteropolyblue was then employed for the preparation of supported metal nanoparticles. For this purpose, the material in suspension in water was first irradiated with a 300-W high-pressure mercury lamp. After 15 min irradiation, the suspension was mixed in dark with a solution of the metal salts (AgNO_3_, HAuCl_4_ or H_2_PtCl_6_). Electronic transfers occurred resulting in a reduction of the metal salt and the formation of metal nanoparticles which were very small in size (several nanometers) and well dispersed on the surface of the polyoxometalate/organic cation/silica composite material.

## 6.Conclusions

As shown in this overview, the field of materials synthesis with entrapped polyoxometalates has been highly diversified during the last years. Starting from a simple hydrolysis and polycondensation of Si(OEt)_4_ in the presence of polyoxometalate, increasingly complex and subtle synthetic methods have been developed over the years leading to a wide variety of materials adapted to numerous applications. The convergence of porous silica synthesis techniques with the highly reactive and tunable class of POM inclusions results in a powerful new tool for functional material design. The emerging field is now being extended to the synthesis of oxides other than silica with encapsulated polyoxometalates, for example POMs/TiO_2_ materials which display interesting properties in photochemistry [56]. Further convergence of these methods with other nanoscale domains (supramolecular species, metal nanoparticles, vesicles …) should provide surprising and interesting applications in the very near future.
